# Case Report: Emergency High-Risk Percutaneous Coronary Intervention Following Transcatheter Aortic Valve Implantation in Bicuspid Anatomy

**DOI:** 10.3389/fcvm.2020.620272

**Published:** 2021-01-20

**Authors:** Ahmed El-Medany, Gemina Doolub, Amardeep Dastidar, Nikhil Joshi, Thomas Johnson, Stephen Dorman

**Affiliations:** ^1^Department of Cardiology, Bristol Heart Institute, Bristol, United Kingdom; ^2^Department of Cardiology, Southmead Hospital, Bristol, United Kingdom

**Keywords:** transcatheter aortic valve implantation, aortic stenosis, percutaneous intervention, case report, coronary angiography

## Abstract

**Background:** Transcatheter aortic valve implantation (TAVR) continues to develop as a valuable alternative to surgical aortic valve replacement (SAVR) in an increasingly wide spectrum of patients with severe symptomatic aortic stenosis (AS). AS frequently coexists with coronary artery disease, and performing technically challenging percutaneous coronary intervention (PCI) following TAVR will become more frequent with increased use of TAVR.

**Case Summary:** We herein report the case of a 53-years-old man with complex medical history including type 1 diabetes and dialysis-dependent renal failure and prior Evolut-R TAVR for critical bicuspid aortic valve stenosis who underwent intravascular ultrasound study (IVUS)-guided PCI to a critical distal left main stem (LMS) and proximal left anterior descending (LAD) lesion after presenting with ventricular fibrillation (VF) secondary to an acute coronary syndrome (ACS).

**Discussion:** Selective engagement of coronary ostia through the side cells of TAVR prosthesis can be challenging, especially in an emergency setting. The particular challenges associated with this case are described, as well as an up-to-date literature search on strategies and equipment that can help in this situation.

## Learning Points

- The need for PCI following TAVR is not uncommon and is primarily related to coronary artery disease progression.
- PCI post-TAVR is usually feasible and safe, although coronary intubation can be challenging due to the superimposition of the metallic frame of the TAVR against the aortic root, particularly in an emergency setting.
- PCI post-TAVR in bicuspid valve anatomy provides unique challenges to coronary access resulting from the higher implant depth.
- The use of guide extension catheters can overcome the catheter intubation challenges.

## Introduction

Since the advent of transcatheter aortic valve replacement (TAVR), its use has been rapidly expanding due to the growing elderly population and concomitant age-related high prevalence of aortic stenosis (AS). The expanding randomized controlled trial evidence base now supports the appropriateness of TAVR, in discussion with the heart team, in inoperable, high-risk, intermediate-risk, and low-risk cases. This has led to an exponential increase in procedural volume (1).

There is considerable overlap between the presence and pathophysiology of atherosclerotic coronary artery disease (CAD) and AS, with significant CAD observed in around half of individuals presenting with severe AS (2), and as TAVR inevitably expands toward the management of younger and lower risk patients (3, 4), the likelihood of encountering potentially significant CAD after TAVR requiring emergency or semi-elective percutaneous coronary intervention (PCI) will increase.

This poses a new technical challenge for the coronary interventionalist, whereby the design, material, and anatomic orientation of various TAVRs may impact the feasibility and safety of coronary angiography (CA) and PCI. The current consensus on the optimal approach to investigation and revascularization in these patients is limited (5). Therefore, we hereby report a case of successful revascularization of complex CAD of the left main stem (LMS) and left anterior descending (LAD) with intravascular ultrasound study (IVUS)-guided PCI.

## Case Description

A 53-years-old man was referred to our institution following cardiac arrest secondary to ventricular fibrillation (VF) while receiving hemodialysis at a nearby hospital. He received immediate cardiopulmonary resuscitation (CPR) and had return of spontaneous circulation (ROSC) following two shocks *via* transdermal defibrillator. His medical history included type 1 diabetes with associated neuropathy, morbid obesity, hemodialysis-dependent end-stage renal failure (ESRF), diffuse coronary artery disease, and multiple sclerosis. Five months earlier, following heart team discussion, he had undergone TAVR with a 26-mm Medtronic Core Valve Evolut R (Medtronic, Minneapolis, MN, USA) for severe symptomatic AS secondary to a Sievers Type 0 bicuspid aortic valve. CA prior to TAVR, 9 months prior to admission, suggested mild LMS disease with severe right coronary artery (RCA) disease, although image quality was poor.

On admission, the patient was alert with a Glasgow Coma Score of 15/15 and asymptomatic. Physical examination revealed a pan-systolic murmur over the apex. Other physical findings included pulmonary rales in the lower lung fields, mild distention of the jugular veins, and mild lower extremity edema. A 12-lead electrocardiogram showed sinus rhythm and biphasic T-waves in the lateral leads. Venous blood gas analysis revealed lactate, pH, and potassium within normal limits. Troponin T was 547 ng/L, rising to 836 ng/L on repeat assessment 10 h later, in the context of end-stage chronic renal failure and a creatinine of 440 μmol/L. Bedside transthoracic echocardiography (TTE) showed long-standing akinesia of the inferolateral segments, with mild left ventricular systolic impairment and inferolateral hypokinesia. Chest X-ray revealed upper lobe blood diversion and cardiomegaly only.

### Timeline

**Table:** 

Day 0	Patient with complex history (as detailed in case presentation) was admitted following cardiac arrest secondary to VF while receiving routine dialysis.
Day 0	Further episode of polymorphic ventricular tachycardia
Day 0	CA performed *via* left femoral artery. Difficulty engaging left and right coronary cusps due to TAVR. Critical LMS, LAD, and intermediate disease were described.
Day 1	Further VF arrest overnight. Proceeded to repeat CA and angioplasty to LMS/LAD. Transferred to intensive care unit (ICU) for hemofiltration considering ESRF and high contrast load.
Day 5	Decision for ICD, as residual CAD was felt to be a potential arrhythmogenic substrate
Day 15	Secondary prevention ICD implanted
Day 21	Discharged home from our institution

### Diagnostic Assessment, Intervention, and Follow-Up

The decision was made to transfer the patient to the coronary care unit (CCU) for observation, where he had a monitored episode of non-sustained polymorphic ventricular tachycardia and subsequently underwent CA. There were difficulties engaging the LMS due to the TAVR cells, although the overall impression was of severe LMS, LAD, and intermediate disease. The operator was unable to comment on RCA disease, as they were unable to selectively engage the RCA. The patient was commenced on dual antiplatelet therapy and planned for multidisciplinary team (MDT) discussion regarding the optimal approach to management of his CAD. Overnight, in the CCU, the patient experienced further VF with loss of cardiac output and required resuscitation. There were no localizing ST segment changes on 12-lead electrocardiography and a repeat Troponin T level was 747 ng/L. He was therefore discussed the following day with the surgical team in view of the clear pattern of diabetic multivessel disease, which was felt to be best treated long term with bypass; however, due to the high surgical risk, the decision was made to pursue percutaneous angioplasty, accepting the risk of incomplete revascularization.

**Figure 1:**
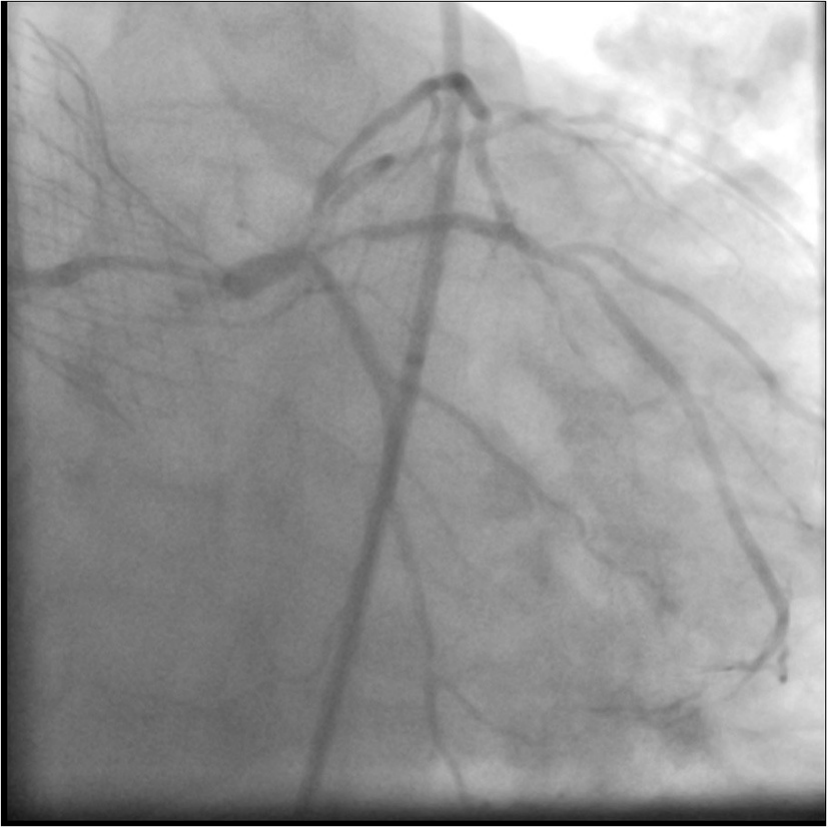
Coronary angiography showing a tapering distal LMS lesion, with critical proximal LAD disease. There is also a severe ostial D1 lesion, severe proximal intermediate disease, and diffuse diabetic disease throughout.

The risks of the procedure were thoroughly discussed with the patient who agreed. CA was undertaken *via* the right femoral artery [ultrasound guided using a 7-French (7F) sheath]. A second experienced PCI and TAVR operator then attempted to selectively cannulate the LMS through the nitinol frame of the CoreValve. Similar difficulties were encountered with a tendency to engage superiorly and non-coaxially due to the high position typical of a bicuspid implant and the orientation of the Evolut commissures relative to the LMS origin. Eventually, selective cannulation was achieved using a 7F JL4 guide catheter and 7F guideliner over a 0.035-inch wire (Supplementary Video 1). CA (Figure 1, Supplementary Video 2) confirmed a tapering distal left main lesion, with critical proximal LAD disease and further severe ostial D1 lesion and diffuse diabetic disease throughout, and severe proximal intermediate disease. The RCA was not selectively intubated but was seen to be open proximally.

In order to clarify the appropriate PCI strategy, the LAD and intermediate vessels were interrogated using IVUS. This demonstrated a 2–2.5-mm intermediate vessel with extensive calcification (360° calcium arc). Decision was made to treat the culprit left main/LAD disease and to avoid a two-stent bifurcation lesion involving the intermediate vessel in view of the long-term risks of stent failure and probable non-viability in the intermediate artery territory (long-standing inferolateral akinesia on TTE). The LAD was predilated using a 2.5-mm balloon with subsequent stent deployment with a 3.5 × 21 mm Ultimaster™ sirolimus-eluting stent (Terumo Interventional Systems) to the distal left main/LAD (Supplementary Figure 1, Supplementary Video 3). Post-dilatation was performed using a 3.5-mm non-compliant balloon to the LAD and 4.5/5-mm balloon to the left main stem.

Kissing inflation was then undertaken to the LAD/first diagonal branch using 2.5/3-mm balloons. An 8F Angioseal was deployed for closure, and the patient was transferred to the intensive care unit (ICU) overnight for filtration. Complete revascularization of his severe diffuse diabetic three-vessel disease was felt to be desirable but impractical without surgical conduits and hence only the culprit lesion was treated. A subsequent MDT confirmed the view that the substrate for ventricular arrhythmia was likely to remain, and so he underwent implantable cardiac defibrillator (ICD) implantation prior to his successful discharge.

## Discussion

TAVR has revolutionized the management of symptomatic severe AS, and indications are expanding toward treating younger and lower risk patients (6). AS and CAD share common risk factors and pathophysiology, and previous studies have shown a 30–50% prevalence of CAD in patients with symptomatic severe AS undergoing conventional surgical aortic valve replacement (SAVR) (7, 8).

There is a paucity of data describing the incidence of coronary events following TAVR, although given the progressive nature of CAD, CA, and PCI will become increasingly necessary in patients after TAVR (9). Therefore, acquisition of appropriate images of the coronary arteries by selective CA is paramount to establish an accurate diagnosis and management strategy. However, the presence of a TAVR bioprosthesis could potentially complicate or inhibit the selective catheterization of the coronary ostia. Existing data, based on isolated cases and small series globally, have assessed the safety and feasibility of CA and PCI following TAVR. Chetcuti et al. (10) reported successful coronary access in 186/190 (97.9%) of their cases but successful PCI in only 103/113 cases (91.2%) following CoreValve TAVR.

**Figure 2:**
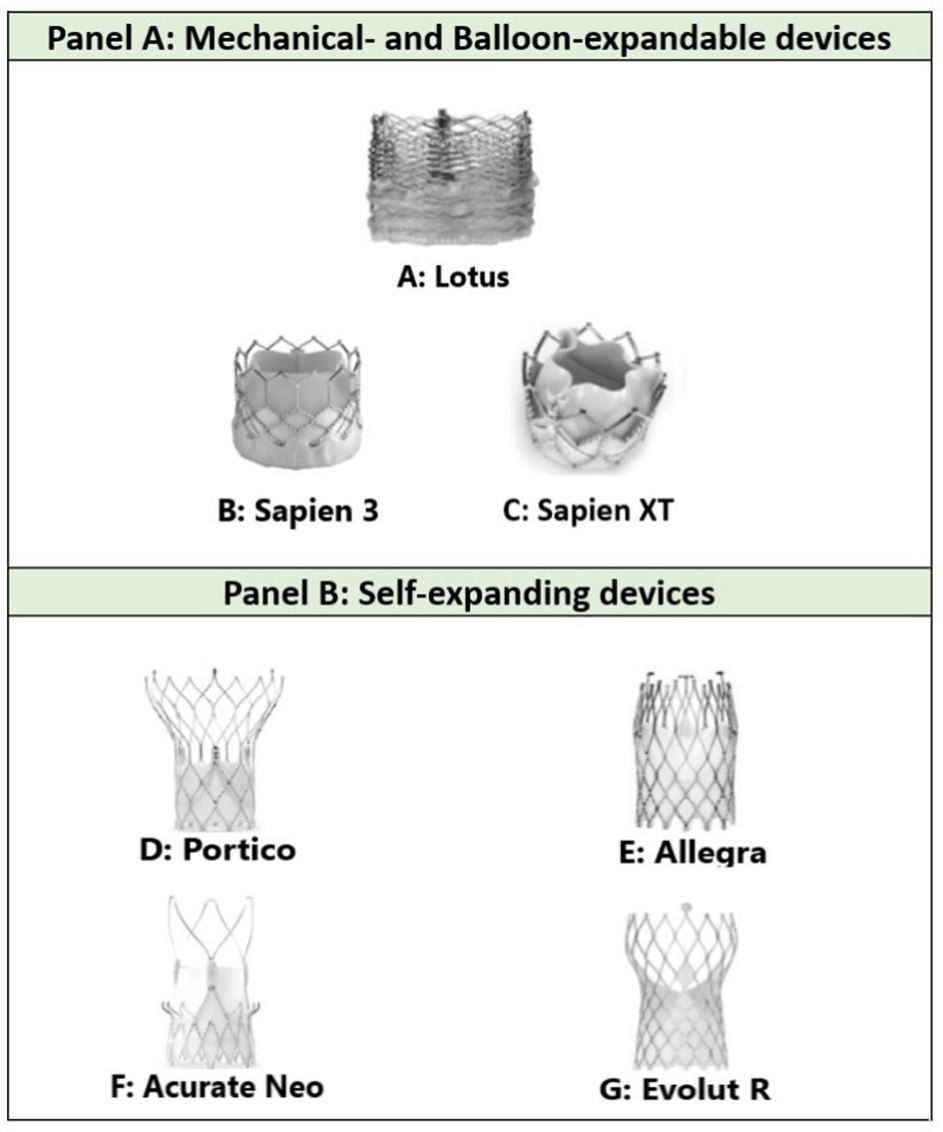
**(A)** The densely packed, horizontally aligned, narrow cells seen in Lotus valves can result in difficulty with intubating the coronary ostia; **(B,C)** the large cells and low density mesh of Sapien valves enable coronary cannulation; **(D)** the large cell area of Portico and its annular positioning facilitates easy coronary engagement although tall frame height can be challenging; **(E)** the shorter stent design and larger cells of the upper section of the Allegra stent frame might allow for easier coronary access; **(F)** Acurate Neo valves display both favorable (short stent component) and unfavorable (tall commissural post) features; **(G)** the relatively tall frame of Evolut R can prove a challenge in coronary angiography, although the presence of a black marker in principle facilitates optimal placement to simplify future coronary access (11, 12). Modified from Todaro et al. (11) and Yudi et al. (5).

The different types of commonly used TAVR present various challenges by virtue of their overall structure and cell size, as illustrated in Figure 2 [modified from Todaro et al. (11) and Yudi et al. (5)].

A large observational study by Zivelonghi et al. (13) compared CA and PCI success rates in both balloon (Sapien 3; *n* = 41) and self-expandable (Evolut-R; *n* = 25) bioprosthesis and concluded that catheterization of the coronary ostia after TAVR was safe and feasible in most cases. However, in 4% of vessels, selective angiography required positioning of a guidewire in the vessel and only one artery could not be engaged due to the high implantation of the Evolut-R, which was hypothesized to be due to the leaflet base of the supra-annular valve landing at the level of the origin of the coronary ostium (13). Tanaka et al. (9) concluded that CA and PCI following the self-expanding CoreValve TAVR are safe and feasible in most cases [28 of 32 (87.5%) LMS angiography successful; 28 of 30 (93.3%) PCI successful], although described difficulty in selective engagement of the RCA [16 of 32 (50%) successful]. The RE-ACCESS study by Barbanti et al. (14) enrolled 300 patients undergoing TAVR using all commercially available devices (Figure 2). The primary endpoint, unsuccessful coronary engagement following TAVR, was demonstrated in 23 of 300 patients (7.7%), which occurred almost exclusively in those receiving Evolut-R devices (9).

With regard to balloon-expandable transcatheter valves, such as the Sapien 3, the main difficulties reported across recent case reports were due to the top of the valve frame partially or completely obscuring the ostium, although this was overcome with the use of either a balloon-assisted tracking or a guide extension catheter (15). With regard to self-expandable valves, such as the Evolut-R CoreValve, the main themes covered by previous case reports and case series include difficulties engaging the coronary ostia and the requirement for different catheters and/or guide extensions to optimize guide support; although despite these challenges, CA and PCI appear feasible, even in complex disease, such as chronic total occlusions (16). The role of post-TAVR computed tomography (CT) can be helpful in determining the anatomy and approach to coronary access; however, it is limited in urgent situations, such as acute coronary syndrome (ACS), and in patients with renal insufficiency who are at increased risk of contrast nephropathy (5).

**Figure 3:**
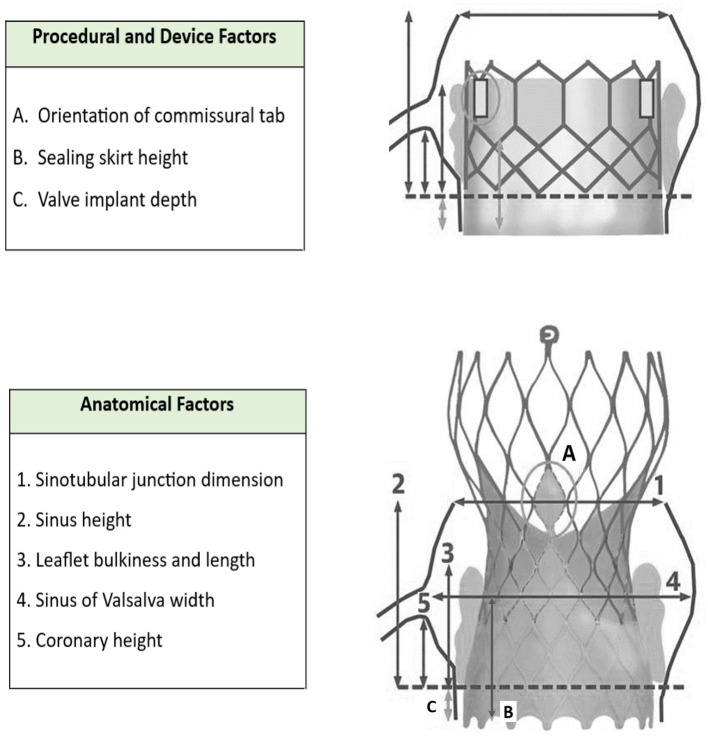
Coronary reaccess following TAVR. Modified from Todaro et al. (11) and Yudi et al. (5).

As illustrated by Figure 3 [modified from Todaro et al. (11) and Yudi et al. (5)], the challenges associated with coronary reaccess following TAVR can be anatomical and procedural.

In order to tackle these particular challenges, Yudi et al. (5) proposed a catheter selection algorithm depending on the type of bioprosthesis (CoreValve, Medtronic, Minneapolis, Minnesota; or Sapien, Edwards, Irvine, California), the type of procedure (CA or PCI), and the position of the transcatheter valve commissure with respect to the coronary ostium. This case nicely demonstrated the utility of a 0.035-inch wire to cross the desired diamond cell and provide a firm rail over which to deliver a suitable guide extension and guide catheter as described in their paper. We would agree that the narrow waist of the CoreValve frame favors the use of the Judkins Left (JL) shape over that of Extra back up (EBU) style for accessing the LMS and hence smaller or short tipped JL catheters or even a Judkins Right (JR) may successfully be employed. We would however disagree with the systematic use of 6F guide catheters in more complex anatomy.

Our case illustrates the limitations of this approach, as typically a guide extension catheter is required to selectively cannulate the coronary ostia in complex coronary anatomy. A 6F guide extension then provides a working internal lumen closer to 5F and so a complex intervention that requires the use of multiple wires, intravascular imaging, or certain calcium modification techniques may not always be safely performed.

Our case also highlights the challenges unique to coronary artery engagement in true bicuspid valve anatomy. Typically, TAVR implants in bicuspid valves preferentially favor a higher deployment that effectively means a greater chance of the sealing skirt of a TAVR valve obstructing direct access to the coronaries. For the CoreValve, this then mandates choosing a higher or more lateral cell to successfully access the coronary ostia. This increases the difficulty of delivery of catheters or equipment due to lack of co-axial alignment. In this scenario, we would strongly advocate the use of guide extensions to enable stents and intravascular imaging catheters to be delivered safely to the coronaries without the risk of entrapment or damage when passing through the cells and around the sinus. Furthermore, with hindsight, we would have considered the implantation of a balloon expandable device, likely the Sapien 3, in the context of high probability of future coronary intervention. Table 1 summarizes the factors to take into consideration when accessing coronaries post-TAVR (5).

**Table 1:** Considerations and approach for PCI post-TAVR (5).

**Consideration**	**Strategy**
Baseline anatomy and TAVR valve type	**TAVR valve type**: • Frame/skirt height, cell size, commissure orientation
	**Baseline anatomy**: • Depth of TAVR implant, baseline aortic root anatomy, coronary height, ascending aorta diameter
Complexity of coronary intervention proposed	Consider need for intravascular imaging, Calcium modification or two-stent strategies
Choice of guide	**LCA**: Preference for smaller short-tipped JL shape over EBU due to narrow aortic root constraints **RCA**: JR4 or usual catheter choice • French size: Guide extension use common and so consider 6F, 7F, or 8F systems depending on complexity of proposed intervention
Access	**Operator preference**: • Radial or ultrasound-guided femoral • Left radial approach may be favored over right radial
Intubating the coronary ostium	**Simple cases**: • Direct intubation • Intubation over coronary guidewire/buddy balloon/balloon tracking • Guide extension
	**Complex cases**: • Use a 0.035-inch J wire to enter closest diamond (ideally in front of coronary ostia). Railroad guide extension, then guide catheter over wire. • Stiff angled glide wire if difficulty persists entering diamond

## Conclusion

CAD remains one of the most frequent comorbidities among TAVR candidates. Scarce data exist on the occurrence, impact, and management of coronary events following TAVR. Although complex PCI is technically feasible following TAVR, this requires an understanding of the anatomy of the valve inserted, the relative implantation depth of the valve, the orientation of the commissures, and the width of the aortic root. In particular, the bicuspid anatomy provides unique challenges that require careful planning. In addition to better understanding the pathophysiology and establishing the most appropriate treatment strategy in such cases, more data are urgently needed regarding the coronary access, particularly feasibility and failure rate, across different transcatheter valve types.

## Data Availability Statement

The raw data supporting the conclusions of this article will be made available by the authors, without undue reservation.

## Ethics Statement

Written informed consent was obtained from the individual(s) for the publication of any potentially identifiable images or data included in this article.

## Author Contributions

AE-M and GD drafted and revised the manuscript and contributed to the conception and design of the case report. AD, NJ, TJ, and SD contributed to the conception of the study and revised the manuscript. All authors approved the final manuscript as submitted and agree to be accountable for all aspects of the work.

## Conflict of Interest

The authors declare that the research was conducted in the absence of any commercial or financial relationships that could be construed as a potential conflict of interest.

## Supplementary Material

Supplementary Figure 1 **Coronary angiography showing 3.5 × 21 mm UltimasterTM sirolimus-eluting stent in the distal LMS/LAD**.

Supplementary Video 1 Coronary angiography showing selective cannulation using a 7F JL4 guide cather and 7F guideliner over a 0.035-inch wire.

Supplementary Video 2 Coronary angiography showing a tapering distal LMS lesion, with critical proximal LAD disease. There is also a severe ostial D1 lesion, severe proximal intermediate disease, and diffuse diabetic disease throughout.

Supplementary Video 3 Coronary angiography showing 3.5 × 21 mm UltimasterTM sirolimus-eluting stent in the distal LMS/LAD.
