# Supplementary figures and images for: Case Report: Emergency High-Risk Percutaneous Coronary Intervention Following Transcatheter Aortic Valve Implantation in Bicuspid Anatomy
Source: Front Cardiovasc Med. 2021 Jan 20;7:620272. doi: 10.3389/fcvm.2020.620272 (PMC7854893; doi:10.3389/fcvm.2020.620272)

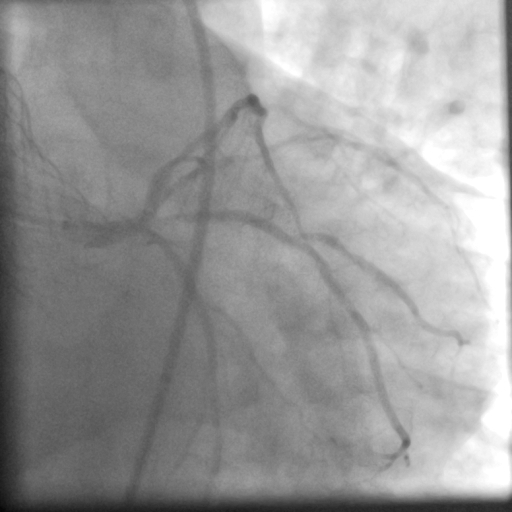

Supplement: Supplementary Figure 1 — Coronary angiography showing 3.5 × 21 mm UltimasterTM sirolimus-eluting stent in the distal LMS/LAD. [file Image_1.JPEG]
